# Virulence evolution of a salmonid virus following a host jump

**DOI:** 10.1371/journal.ppat.1013806

**Published:** 2025-12-17

**Authors:** Malina M. Loeher, Gael Kurath, David A. Kennedy, Joanne E. Salzer, William N. Batts, Rachel B. Breyta, Andrew R. Wargo

**Affiliations:** 1 Virginia Institute of Marine Science, William & Mary, Gloucester Point, Virginia, United States of America; 2 U.S. Geological Survey, Western Fisheries Research Center, Seattle, Washington, United States of America; 3 The Pennsylvania State University, University Park, Pennsylvania, United States of America; 4 University of Washington, Seattle, Washington, United States of America; University of North Carolina at Chapel Hill, UNITED STATES OF AMERICA

## Abstract

Emergent viral diseases remain a critical obstacle to welfare across landscapes and species, encompassing humans, wildlife, and agriculture. Following a jump to a novel host, the severity of disease resulting from infection is a critical determinant of the overall emergent pathogen threat. Conventional wisdom posits that virulence, defined here as host mortality, attenuates to intermediate levels as a pathogen adapts to a novel host, but this is largely based on data from just one system, myxoma virus, which was intentionally introduced as a biocontrol agent in rabbits (*Oryctolagus cuniculus*) in mid-1900s Australia. In this study, we demonstrate that infectious hematopoietic necrosis virus (IHNV), which made a host jump from sockeye salmon (*Oncorhynchus nerka*, ancestral host) to rainbow trout (*O. mykiss*, novel host), has not conformed to classical theory. We quantified virulence in the ancestral and novel hosts using common garden *in vivo* experiments with 16 archival IHNV isolates collected from 1972-2017, which span the period from shortly after the host jump and the subsequent 45 years of host adaptation. These virus isolates also represent two distinct phylogenetic genogroups, each associated with either the ancestral or novel host. The experiments were replicated across two research facilities, two challenges dosages, and two temperatures. While isolates from the ancestral genogroup showed no temporal change in virulence in either host, isolates from the novel viral genogroup displayed a significant increase in virulence over time in the novel host. Some possible indication of a virus temperature adaption after the host jump was also present. Potential drivers of virulence evolution are discussed. This represents one of only a handful of systems in which the evolution of increased virulence has been empirically characterized after a host jump and subsequent adaptation. It contributes to a growing body of evidence that contradicts the classical case study of myxoma virus attenuation after adaptation.

## Author summary

Conventional wisdom ascertains pathogens will become less harmful as they adapt to a novel host. However, observations across recent pathogen emergence events such as HIV from monkeys to humans or SARS-CoV-2 from bats to humans, do not support this paradigm. Another contrasting system is infectious hematopoietic necrosis virus (IHNV), which emerged in fish farming following a host jump into rainbow trout (*Oncorhynchus mykiss*) from its ancestral host sockeye salmon (*O. nerka*) in the late 1960s. The virus is now one of the primary sources of production losses for trout farming worldwide, and poses serious challenges to conservation. Due to this history and extensive surveillance, IHNV in salmonid fish offers an ideal system to test the theory of pathogen virulence evolution after a host jump. We measured disease severity in the ancestral and novel hosts for 16 viral isolates that represent genetic diversity across five decades. We observed a trend of increasing disease severity among isolates from the novel rainbow trout host, but no temporal trend among isolates associated with the ancestral host. This work demonstrates the potential for increased disease across host jumps, contradicting textbook knowledge. Understanding evolutionary trajectories for emergent pathogens will better focus limited resources towards systems predicted to have health threats.

## Introduction

Emergent viruses constitute a major threat across species and ecosystems. Virulence, here defined as host mortality as a direct result of infection, is the most obvious and urgent consequence of viral emergence. The direction of viral virulence evolution is a critical determinant for assessing the long-term severity of an emergence event, but the capacity to infer this trajectory remains limited [1]. Contemporary virulence evolution theory postulates that increasing virulence diminishes virus transmission duration due to host mortality, and thus has a fitness cost. However, increased virulence is also believed to provide transmission benefits of greater viral replication and host immune system evasion [2–4]. This creates what has been termed the virulence tradeoff, which was classically demonstrated by myxoma virus in Australia’s naïve rabbit population [3,5,6]. In mid-1900s Australia, myxoma virus was used as a biocontrol measure for invasive rabbits, where it had decimating effects in the early years after its release. However, after several decades, myxoma virulence attenuated to intermediate levels, which, through robust common garden experiments, was shown to provide optimal viral fitness [7,8]. The myxoma story was so compelling that it became the textbook example of virulence evolution after a host jump. Subsequently, the possibility that pathogens may evolve decreased virulence after emergence became commonly accepted [9]. However, this hypothesis, which rests on the assumption that pathogens emerge with suboptimal high virulence, has been challenged [4,10].

Virulence evolution in the context of trade-off theory is an area that has received a great deal of research attention, particularly in mathematical-modeling studies (reviewed in [11]). Nonetheless, few empirical studies exist beyond myxoma that investigate how viral pathogens may adapt and evolve following a host jump, and those that do offer contrasting conclusions [3]. According to tradeoff theory, the directionality of virulence evolution is determined by the position of a virus on its respective fitness-virulence curve at the time of emergence, allowing for the possibility of increased virulence in the new host. However, there is little empirical evidence of this trend, in part due to sampling bias where initially benign pathogens are less likely to be detected and reported [3]. A recent non-virus example of gained virulence following emergence is *Mycoplasma gallisepticum* bacterium in North American house finches (*Haemorhous mexicanus*), which evolved increased virulence over two epidemics spanning the mid-1990s to 2010s [12]. Higher *M. gallisepticum* loads and subsequently increased transmission suggest that higher virulence may confer greater overall fitness and be an adaptive trait for some emerging pathogens [12–14]. Indeed, even in the case of myxoma, the virus stabilized at intermediate virulence and did not become completely benign [3–7]), further indicating that virulence could be adaptive.

In addition to these direct experimental approaches, several observational and epidemiological studies have tracked viral virulence evolution after emergence. Systems such as Ebola virus in humans and feline calicivirus in domesticated cats, suggest the evolution of increased virulence over time [15–17]. For HIV, there is evidence that the virus evolved increased virulence in some countries and decreased virulence in others since it emerged in humans, with postulated mechanisms by which intermediate virulence could maximize viral fitness [10,18]. The evolution of emergent SARS-CoV-2 has been an area of high public concern with some evidence that the virus initially increased in virulence [19], and despite the circulation of less virulent variants such as Omicron, the long-term virulence trajectory remains uncertain [19–21]. Yet these studies all face the same limitation; confounding variables such as changes in treatments, interventions, and host immunity make it difficult to quantify how virulence has changed over time [22]. For example, in the case of the SARS-CoV-2 pandemic, most individuals developed some level of protective immunity (either as result of natural infection or vaccination) before the emergence of the Omicron variant. Although Omicron is more adept than earlier variants at infecting human hosts with residual immunity, this prior immunity (in addition to improved therapeutics such as Paxlovid and targeted healthcare), causes individuals to experience less disease, creating a perception of reduced virulence [22]. The more appropriate evolutionary assessment of virulence would be the level of disease caused by Omicron in comparison to previous variants, in non-immune hosts without intervention. Such comparisons are exceedingly difficult in nature, where hosts adapt alongside pathogens and withholding treatment is unethical.

Collectively, the available research indicates that the evolutionary arc of attenuation after emergence found in the textbook case of myxoma cannot be applied to all pathogens. Novel interactions between hosts and emergent pathogens may lead to highly variable health outcomes [23]. As such, additional empirical studies are warranted to determine if generalities can be found, or what specific mechanisms might be most important for driving virulence evolution. The trajectory of virulence evolution after pathogen emergence remains difficult to test. A common limitation is that concurrent changes in host genetics, the environment, or management that occur alongside viral evolution can mask changes in virulence [24,25]. Conclusive evidence requires experiments examining the virulence of multiple viral isolates spanning the host jump and the period that follows, while controlling for host genotype and immune status, and environmental conditions (i.e., common garden experiments). These were features of the myxoma virus studies, but few other systems allow for such investigations.

Here we use one such rare system from an aquatic host to investigate virulence evolution after a host jump: infectious hematopoietic necrosis virus (IHNV). IHNV is a negative-sense, single stranded RNA rhabdovirus in the genus *Novirhabdoviridae* (species *Novirhabdovirus salmonidae*) that can infect many members of the fish family Salmonidae [26]. It is one of the most important pathogens hindering salmonid conservation and aquaculture worldwide, with mortality during outbreaks often reaching up to 50–95% [27–32]. Historical records indicate that IHNV has been endemic in sockeye salmon (*Oncorhynchus nerka*) for well over a century in the Pacific Northwest of North America and continues to circulate as the ancestral U genogroup of virus [32–35]. A new genogroup of IHNV, classified phylogenetically as M [35], arose following a host jump of U virus from sockeye salmon into rainbow trout (*O. mykiss*) in the late 1960s within aquaculture [30,36–38]. The M genogroup virus then evolved and spread in the southern Idaho trout farming region, which rears fish at the warmer temperature of 15°C and expanded commercially in the 1970s-80s [39,40]. M viruses have been shown to be more virulent than ancestral U viruses in rainbow trout, while U viruses are more virulent than M in the ancestral host, sockeye salmon [41,42]. Previous studies also demonstrate that within the M genogroup more virulent isolates have greater infectivity, replication, and transmission potential than less virulent isolates, indicating that high virulence may be adaptive in this system [4,43–47]. Historical records also indicate that the IHNV host jump was likely followed by a temperature adaption [48]. Early observations indicate that IHNV epidemics in rainbow trout occurred for several years at a maximum of 10°C, but were only later reported at the higher water temperature of 15°C in a major trout farming region in Idaho [35,41,48]. Greater virulence has been experimentally demonstrated for one M isolate at 15°C compared to 10°C [41]. However, few studies have tested IHNV in the same host species at both 10 and 15°, which are commonly used as typical environmental temperatures for sockeye salmon and farmed rainbow trout, respectively [41]. In studies with representative U and M viruses, infectivity of U is higher than M in sockeye salmon at 10°C, and M is higher than U in rainbow trout at 15°C [42,46,49]. Temperature adaptation remains an important evolutionary question as it provides insights into under what environmental conditions virulence is most pronounced [29]. IHNV has continued to cause epidemics in Idaho rainbow trout farming facilities [38], and spread globally through aquaculture activities [29,34]. Among the five global genogroups of IHNV [34] the U and M groups in North America have evolved as specialists with high fitness and virulence in sockeye salmon and rainbow trout, respectively [33,35,41,42,46,49,50].

Due to the important economic, ecological, and cultural status of wild and farmed salmon and trout hosts [51–54], intensive efforts have been put into IHNV surveillance, yielding an archive of thousands of virus isolates [55]. Using a collection of 16 U and M genogroup isolates collected across time following the host jump from 1974-2017, we conducted *in vivo* experiments in the ancestral and novel hosts across two research facilities, two challenge dosages, and two temperatures, to quantify how virulence evolved over the five decades following the host jump.

## Results

### Survival kinetics trends

**Fig 1:**
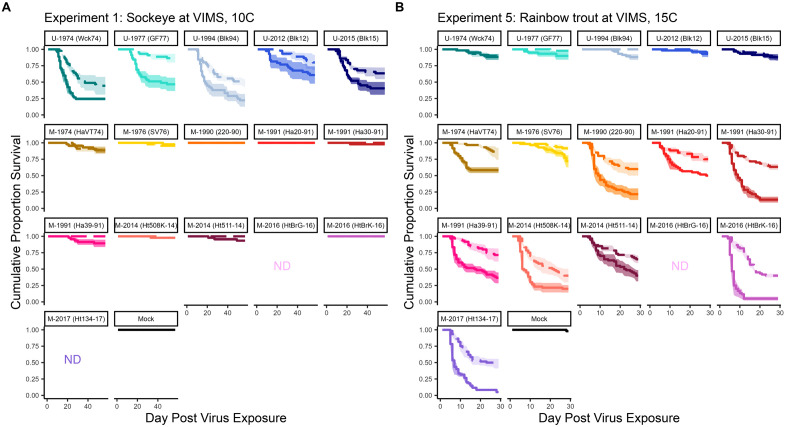
Illustrative cumulative survival data from select virulence assays. Panels show mean cumulative proportion survival through time, for triplicate tanks of 20 fish in each experimental treatment. **(A)** Experiment 1; sockeye held at 10°C at VIMS. **(B)** Experiment 5; rainbow trout held at 15°C at VIMS. Solid lines indicate high dose (2 x 10^5^ pfu/mL); dashed lines indicate low dose (2 x 10^3^ pfu/mL) virus exposure. Standard error (± 1) between the triplicate tanks is indicated by a shaded ribbon. Treatments that were not included in an experiment are marked ND. Treatment plots are ordered by IHNV genogroup (U top row, M bottom rows), followed by year of isolation and isolate name. Mortality was tracked for longer in sockeye experiments so x-axis scales are different. Studies were also replicated at 10°C in rainbow trout and a second facility (the WFRC), resulting in four additional experiments (2, 3, 4, and 6), the data from which is shown in the Supplement.

For the novel rainbow trout host, mortality peaked at 5–10 days and plateaued between days 10–14 post-exposure to virus in duplicated controlled laboratory experiments (Figs 1, S1). The kinetics of mortality were slightly faster at 15°C compared to 10°C, as well as for emergent M compared to ancestral U genogroup isolates. For the ancestral sockeye salmon host, mortality peaked between days 10–15, then continued at a steady rate until slowing between days 20–25. Novel M genogroup isolates caused virtually no mortality among ancestral sockeye hosts, and in the few cases where it was observed, mortality occurred later than for ancestral U genogroup isolates. Within each host species and viral genogroup, there was substantial variation between isolates in survival kinetics, with more virulent isolates (measured as cumulative mortality) typically causing more acute mortality with a clear peak incidence period, and less virulent isolates resulting in more protracted mortality. Survival kinetics patterns were consistent across experiments duplicated at two different laboratories, except for one experiment (experiment 6, S1 Fig) generally having higher mortality at the WFRC than its paired experiment at VIMS (experiment 5, Fig 1B). To account for these effects, location was included as a random term in all subsequent analyses. In all challenges, no mortality occurred for fish exposed to the mock treatment in the first three weeks, by which time most survival curves had plateaued in virus exposed fish. Some mortality (19%) was observed in the 15°C experiment at the WFRC on days 11–27 in the mock tanks, but this was much lower and later than the kinetics observed in virus treatment tanks. This mortality equated to seven individual fish, four of which were titered for IHNV, and two of which tested positive. The positive fish came from a single tank on Days 19 and 24, indicating very low level (<1%) cross contamination occurred either between tanks or during sample processing [4,45].

### Evolution of M virulence through time in novel host rainbow trout

**Fig 2:**
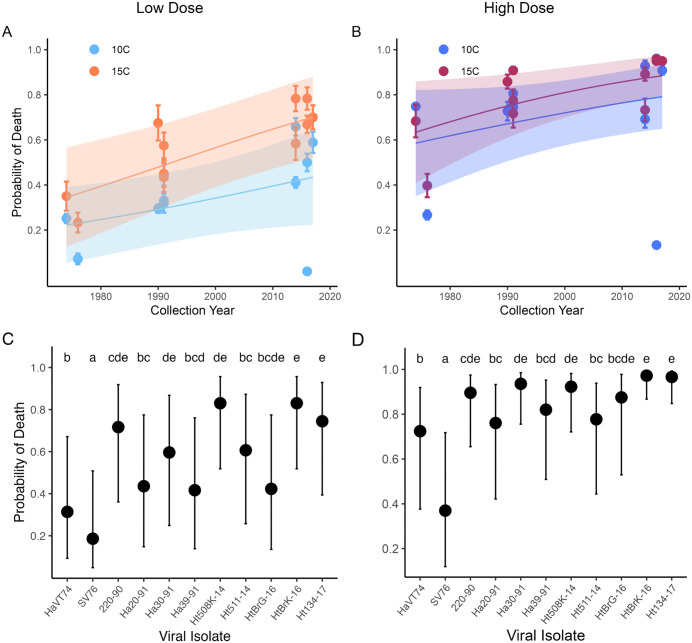
Virulence evolution and variation of M genogroup isolates through time, by dose, in the novel rainbow trout host. **(A,B)** Lines show predicted probability (±95% confidence interval: shading) of fish death as a function of year of viral collection, obtained from AICc-selected statistical models (see methods and S1–S2 Tables) for 15°C and 10°C for low (A - 2 x 10^3^ pfu/mL) and high (B - 2 x 10^5^ pfu/mL) virus exposure dosage experiments in rainbow trout. Points show mean raw data across six replicate tanks (WFRC and VIMS data combined) with standard error bars. Although the dosages are shown separately, the analysis indicated that there was no dose interaction with year, so the slopes of the lines across years and within temperatures are the same for both dosages, in the untransformed logit scale. **(C, D)** Estimated marginal means of predicted probability of fish mortality for M isolates when compared directly to one another in rainbow trout at 15°C at low (C - 2 x 10^3^ pfu/mL), and high (D - 2 x 10^5^ pfu/mL) virus exposure dose, ordered by collection year, obtained from AICc-selected models (see methods and S3–S5 Tables). Letter symbols are comparable within but not between panels C, D and indicate statistical differences at p < 0.05 if a letter is not shared between points (S3–S6 Tables). Bars represent 95% confidence intervals. Refer to Table 1 for Isolate information.

**Fig 3:**
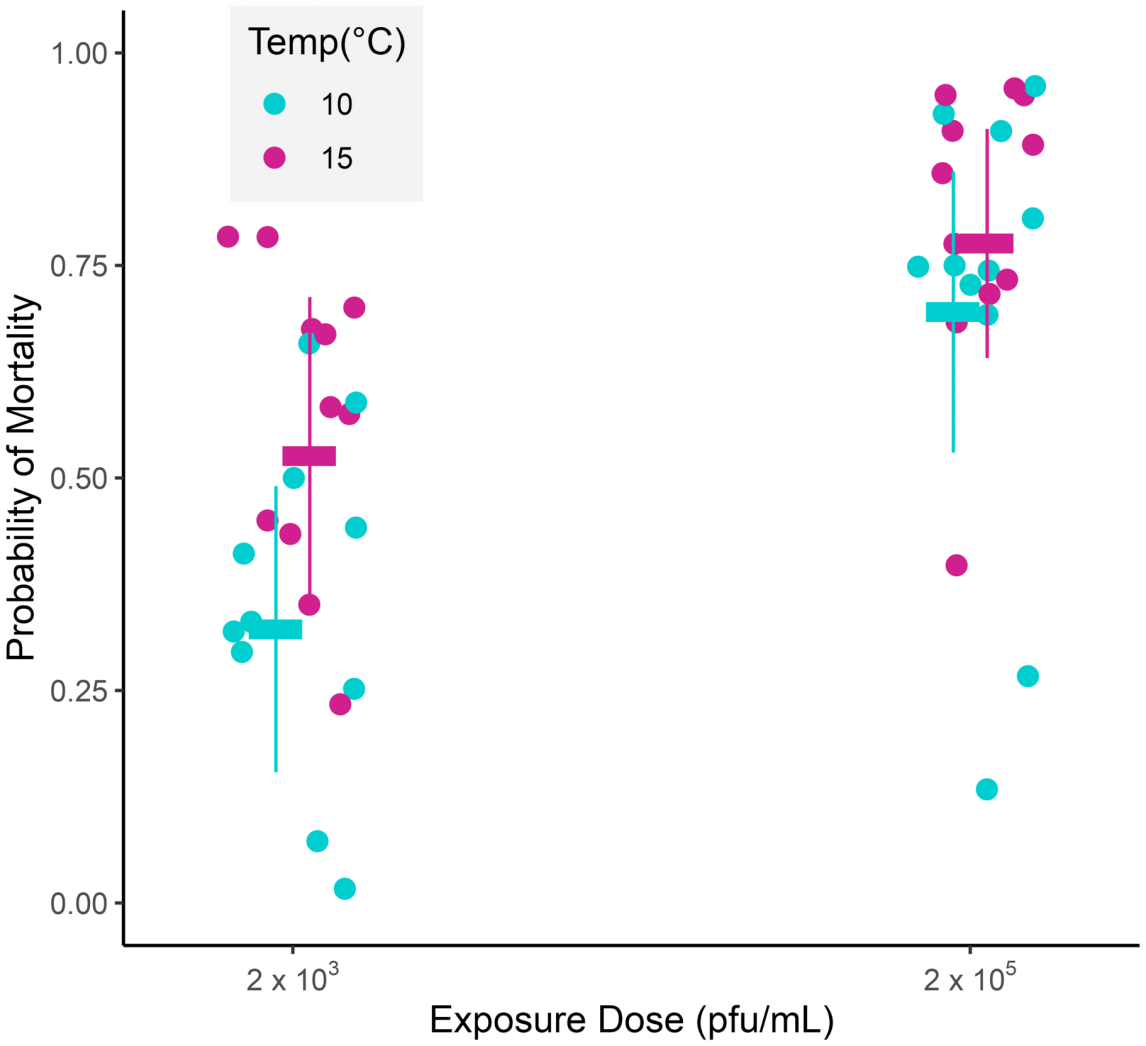
Dose by temperature interaction for M genogroup evolution. Predicted probability of cumulative percent mortality (horizontal bars, jittered) for M genogroup isolates in rainbow trout hosts at different doses (2 x 10^3^ or 2 x 10^5^ pfu/mL) of IHNV at two temperatures (10°C in cyan or 15°C in magenta). Mean raw experimental data across tanks and sites (solid circles, jittered) are shown for all isolates. Predicted data are obtained from selected AICc models (see methods and S1–S2 Tables), plotted with 95% confidence intervals (vertical whiskers).

M genogroup IHNV isolates caused cumulative mean mortality proportions ranging from 0.1 to 1.0 in rainbow trout (Figs 1B, S1). The temporal analysis revealed that for M isolates in rainbow trout, every unit increase in virus collection year conferred an average increase of 2.3% in the odds of death [95% CI: increase of 0, 5.1%] (Fig 2, ΔAICc = 2.70, S1 and S2 Tables). In other words, the most recent M isolates (collected 2014–2017) caused substantially greater mortality than older isolates (collected 1974, 1976) in the novel host. This trend was even more pronounced for fish reared at 15°C, such that the average increase in the odds of death per year was 150% greater [CI: increase of 88, 230%] compared to fish reared at 10°C (Fig 2, ΔAICc = 0.90, S1 and S2 Tables). Therefore, the difference in virulence between new and older isolates was larger in experiments conducted at 15°C compared to 10°C. In general, the higher viral exposure dose resulted in a greater odds of death than the low dose regardless of viral isolation year. However, the dose effect was more pronounced at 10°C compared to 15°C (Fig 3, dose * temp interaction, ΔAICc = 1.8, S1 and S2 Tables). This interaction also resulted in a significantly higher odds of fish death at 15°C compared to 10°C at the low dose, but a proportionally smaller temperature effect at the high dose. Ultimately, when directly comparing the isolates to each other, the highest predicted probability of death (0.97 high dose, 0.83 low dose) was from the second-most recent emergent M isolate (HtBrk-16, collected 2016) and the lowest probability of death (0.37 high dose, 0.19 low dose) from the second oldest isolate (SV76, collected 1976), at the most biological relevant temperature of 15°C (Fig 2C, 2D, S3–S6 Tables).

**Table 1:** IHNV Isolate information.

Label	Isolate Name	Collection Location	Collection Year	Species of Isolation	Genogroup	Subgroup	Sequence type
1	Wck74	Weaver Creek, B.C.	1974	*O. nerka*	U	UP	mG004U
2	GF77	Glacier Flats, AK	1977	*O. nerka*	U	UP	mG003U
3	Blk94	Baker Lake Fish Hatchery, WA	1994	*O. nerka*	U	UP	mG002U
4	Blk12	Baker Lake Fish Hatchery, WA	2012	*O. nerka*	U	UP	mG050U
5	Blk15	Baker Lake Fish Hatchery, WA	2015	*O. nerka*	U	UP	mG265U
6	HaVT-74	Hagerman Valley, ID	1974	*O. mykiss*	M	MN	mG400M
7	SV76	Mission, B.C.	1976	*O. mykiss*	M	MN	mG401M
8	220-90	Hagerman Valley, ID	1990	*O. mykiss*	M	MB	mG009M
9	Ha20-91	Hagerman Valley, ID	1991	*O. mykiss*	M	MB	mG079M
10	Ha30-91	Hagerman Valley, ID	1991	*O. mykiss*	M	MC	mG119M
11	Ha39-91	Hagerman Valley, ID	1991	*O. mykiss*	M	MD	mG107M
12	Ht508k-14	Hagerman Valley, ID	2014	*O. mykiss*	M	MB	mG296M
13	Ht511-14	Hagerman Valley, ID	2014	*O. mykiss*	M	MD	mG298M
14*	HtBrG-16	Hagerman Valley, ID	2016	*O. mykiss*	M	MB	mG342M
15	HtBrK-16	Hagerman Valley, ID	2016	*O. mykiss*	M	MB	mG331M
16**	Ht134-17	Hagerman Valley, ID	2017	*O. mykiss*	M	MC	mG335M

Rows list IHNV isolates used in the study obtained from WFRC freezer archive and originally collected from the field, with accompanying metadata. For isolate name, letters typically signify location, and last two numbers date of isolation. Phylogenetic genogroup, subgroup, and sequence type were determined by mid-G gene sequencing. *Isolate 14 was not tested in VIMS experiments. **Isolate 16 was not tested in sockeye hosts.

### Comparison of M to U virulence

**Fig 4:**
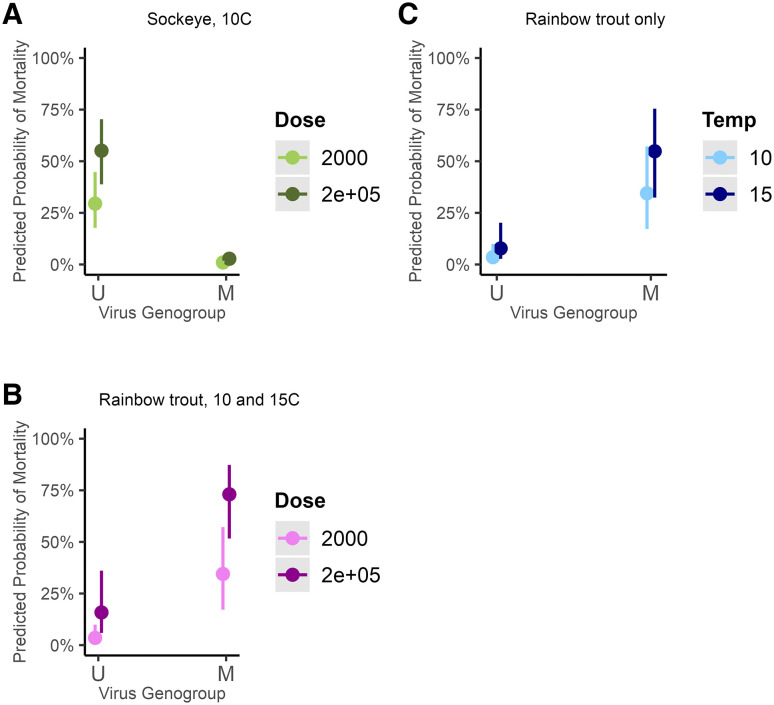
Predicted probability of mortality between genogroups within hosts. Panels A and B show main dose and genogroup effects respectively for sockeye salmon at 10°C, and rainbow trout at 10°C and 15°C, combined. Despite suggestive trends, there was no significant interaction between dose and genogroup for either fish species in the top model (S7–S8 Tables). Panel C shows the genogroup and temperature main effects for rainbow trout hosts. Again, despite suggestive trends, there was no significant interaction between the terms in the top model (S9–S10 Tables). For all panels, bars are 95% confidence intervals. No other interactions or factors were found to be significant in the analyses.

For ancestral sockeye salmon hosts, ancestral U genogroup isolates consistently caused high levels of mortality while emergent M genogroup isolates caused low or no mortality (Figs 1A, S1). The cumulative odds of sockeye mortality increased by 4206% [CI: increase of 1682, 10305%] when fish were exposed to a U isolate compared to an M isolate (Genogroup main effect, ΔAICc = 22.58, S7 and S8 Tables), with model-estimated probabilities of death ranging from 18-70% versus 1–5% respectively (Fig 4A). Sockeye in the higher dose treatment were also more likely to die compared to the lower dose treatment, such that the odds ratio increased by 194% [CI: increase of 124, 284%] (Dose main effect, Fig 4A, ΔAICc = 62.78, S7 and S8 Tables).

Novel rainbow trout hosts displayed the opposite relationship compared to sockeye; emergent M isolates increased the odds of host death by 1340% [CI: increase of 456, 3630%] compared to ancestral U isolates (Genogroup main effect, Fig 4B–4C, ΔAICc = 14.69, S9 and S10 Tables). The predicted probability of rainbow trout mortality was 1–36% versus 17–87% for U compared to M genogroup isolates respectively (Fig 4B-C). As in sockeye hosts, the higher dose treatments in rainbow trout resulted in an increased odds ratio of host death by 415% [CI: increase of 310, 547%] compared to low dose treatments (Dose main effect, Fig 4B, ΔAICc = 164.88, S9 and S10 Tables). The analysis also indicated that rainbow trout were more susceptible to mortality at 15°C compared to 10°C, which increased the odds of death by 131% [CI: increase of 84, 189%], regardless of the virus genogroup (Fig 4C, Temperature main effect, ΔAICc = 47.83, S9 and S10 Tables). Although not directly tested, mortality was qualitatively higher for U isolates in rainbow trout compared to that of M isolates in sockeye (Figs 1, 4).

### Variation in virulence among U isolates in ancestral host sockeye salmon

**Fig 5:**
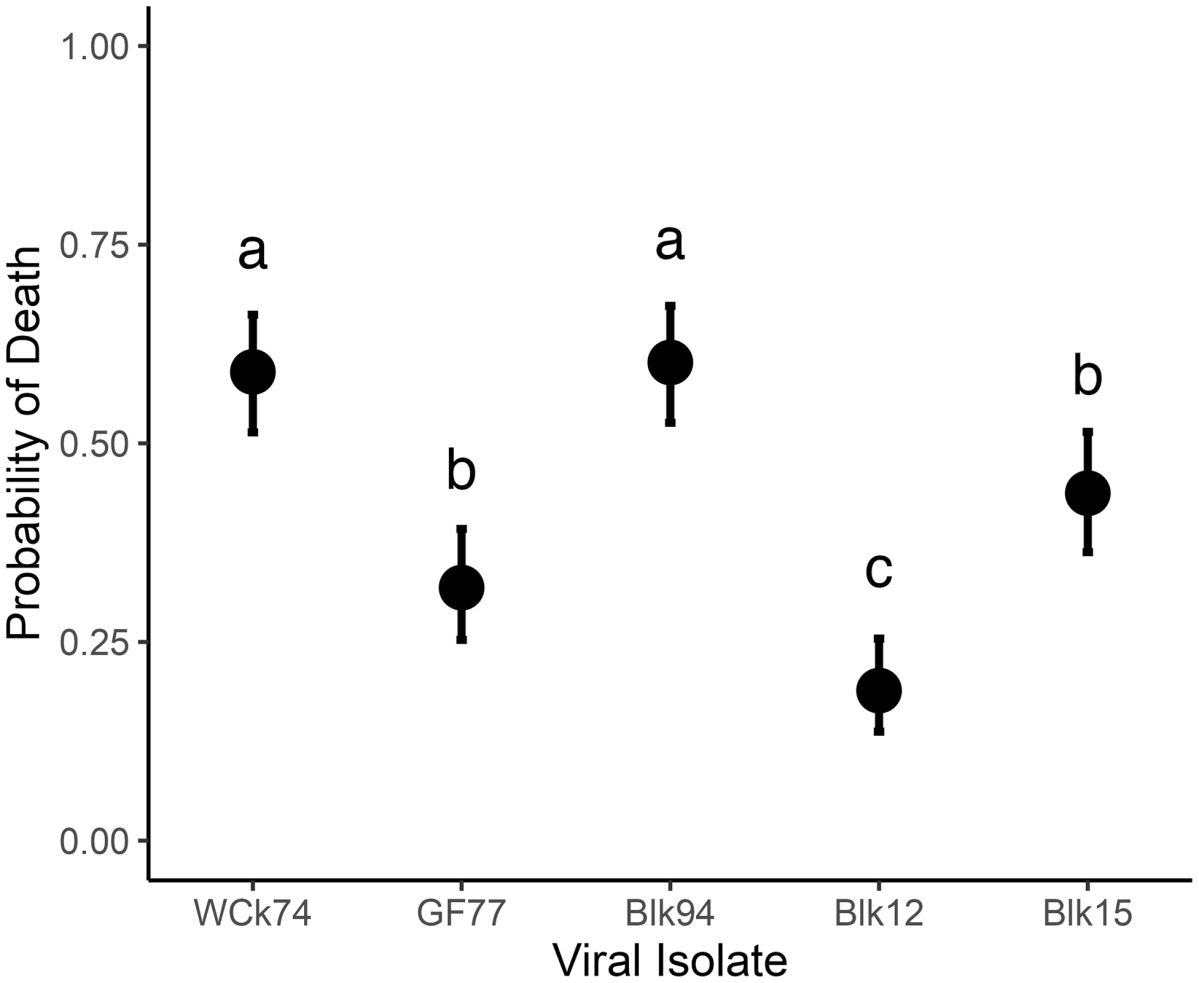
Probability of fish mortality in ancestral host and virus. Points shown predicted probability of fish mortality for U isolates in sockeye salmon at 10°C, obtained from AICc selected models (see methods and S11 Table). Differing letter symbols indicate statistical differences at p < 0.05 (S11 Table). Bars represent 95% CI. No interaction was observed between isolates and dose so data are averaged over dose levels.

Overall, ancestral U isolates incurred mean cumulative probability of mortality ranging from 0.19 to 0.60 in the ancestral sockeye salmon host (Figs 1A, S1). The odds of fish death significantly differed between U isolates, with Blk12 being the least virulent and isolates Wck74 and Blk94 being the most virulent (Fig 5, Isolate main effect, ΔAICc = 80.2, S11 and S12 Tables). Isolates GF77 and Blk15 caused moderate levels of mortality, the odds of which was significantly different from the other three isolates but not from each other. As such, there was no indication of a temporal trend of year of virus isolation for the virulence of U genogroup isolates in sockeye ancestral hosts. An effect of dose was observed, such that the odds of fish death increased by 207% [CI: increase of 129, 311%] in treatments which received the higher dose of viral exposure relative to the lower dose (Dose main effect, ΔAICc = 56.8, S11 and S12 Tables).

## Discussion

Collectively, our results indicate that emergent M genogroup IHNV rapidly gained trout-specific virulence after a host jump from sockeye salmon into rainbow trout, and then continued to increase in virulence through time. Consequently, the most recently collected M isolate of IHNV was on average 1.4 times more likely to cause death in the novel rainbow trout host than those collected in the 1970s (probability death present/probability death past, Fig 2A-B), which appear to have suboptimal virulence. These findings are supported by our six independent studies of sixteen viral isolates temporally spanning time from soon after the host jump event to present day; replicated across the ancestral and novel salmonid hosts, two research facilities, two viral exposure dosages, and two temperatures. Our results therefore offer a distinct contrast to the textbook case of myxoma virus in Australian rabbits [8] where initially high virus virulence attenuated over time following a host jump [9]. The evolution of IHNV towards increased virulence observed here, alongside previous work [4,43–47], suggests that high virulence is either directly or indirectly adaptive for the virus. These results agree with a growing body of literature from systems such as Ebola, feline calicivirus, HIV, SARS-CoV-2, and *M. gallisepticum*, which provide evidence of the evolution of increased pathogen virulence after host jump events [12,13,16,17,19].

The question remains as to what system properties drive virulence evolution and whether generalities can be reached. We note that in the most famous study of virulence evolution, myxoma virus in Australia, an anthropogenically introduced strain was specifically chosen for its extreme virulence, potentially priming the virus for attenuation [56]. For *M. gallisepticum*, virulence evolution appeared to be driven in part by anthropogenically supplemented feeding behavior which encouraged repeated host contacts in static locations, facilitating increased virulence [13]. In the case of SARS-CoV-2, immune evasion and routes of transmission modulated by human behavior heavily influenced which strains became dominant in the early months of the COVID-19 pandemic [20,22]. Similarly, anthropogenic drivers of virulence have been observed in other systems, such as the evolution of increased virulence in Marek’s disease attributed to vaccination and poultry farming intensification [57]. Anthropogenic influences should be examined in the context of virulence and transmission relationships to determine whether common practices promote a particular evolutionary trend.

What role anthropogenic drivers played in IHNV virulence evolution, as observed in other systems, is a pertinent question. Past work determined virulence of IHNV is highly variable and driven by factors such viral genotype and dosage, host species and age, and environmental factors such as temperature and rearing conditions [29,41,58,59]. Field observations and previous empirical investigations have indicated high and potentially increasing virulence of the M genogroup viruses circulating in trout farms since the late 1970s [30,38,42,43,46,47]. Aquaculture is one driver theorized to create novel selection opportunities for increased virulence evolution not seen in less intensive conservation-based fish culture or wild ecosystems. Practices such as higher rearing density, accelerated growth rates, genetically homogenous host populations, vaccination, overlapping age cohorts among hosts, and landscape fragmentation have been suggested to select for high virulence [1,30,38]. Many of these factors are common features of the intensive trout farming region in southern Idaho where M genogroup IHNV evolved after the host jump [37,39]. In other salmonid aquaculture systems, intensive farming practices have been linked to increased virulence among newly emergent parasites and pathogens, including *Flavobacterium columnare* bacterial strains and *Lepeophtheirus salmonis* salmon lice, and it has been suggested for infectious salmon anemia virus [60–62].

Our results parallel the evolution of increased virulence in other genogroups and subgroups of IHNV found in regions of *O. mykiss* (rainbow or steelhead trout) culture. In North America the MD subgroup of the M genogroup emerged in steelhead trout (anadromous *O. mykiss*) in conservation hatcheries after a spillback in approximately 1995, and sequentially dominant MD genetic types have been shown to have increasing virulence over time [31]. Following geographic translocation of M genogroup virus to Europe in the 1980s the virus evolved into the E genogroup, which causes widespread disease burden in European rainbow trout aquaculture [63,64]. Among E isolates circulating in Italy, rapid increases in virulence have been documented in two different genetic subgroups, where virulence is positively correlated with viral replication and emergence time rather than genetic clustering [65]. In the 1970s the introduction of U genogroup IHNV to Japan via a shipment of sockeye salmon eggs led to a second, independent host jump from sockeye into rainbow trout aquaculture, resulting in the J genogroup of IHNV. Studies of J isolates circulating in Japan demonstrated increased virulence across collection dates but again, the shift in phenotype did not correlate with genetic subgroups [66]. Whether M genogroup IHNV circulating in North American rainbow trout aquaculture is moving towards an unknown virulence endpoint or stable equilibrium as predicted after a host-jump [67], is unknown. Links between the implementation of specific farm practices and the evolution of IHNV have not been explored, but could shed light on the drivers of virulence and warrant further investigation [1]. We note that among the most recent isolates assayed in this study (2014–2017), some variation in virulence was observed, but the most recent isolate (Ht134-17) was also one of the most virulent (Fig 2C, 2D). This indicates IHNV virulence in North America may not have yet stabilized and may have more evolutionary space to explore.

The role of rainbow trout ecology in shaping emergent M genogroup virulence evolution may be further supported by our finding that the U genogroup did not evolve increased virulence over time (1974–2015) in the ancestral host (Fig 5). The U genogroup in its sockeye host is hypothesized to be an ancient co-evolved host-pathogen relationship [35] having developed in a very different host ecology, in natural settings outside of aquaculture. Given this long evolutionary history, selection over the last 50 years was not expected. An interesting finding was the high variability of U genogroup virulence, indicating that even this ancestral lineage of the virus has potential to explore evolutionary space. Anthropogenic impacts have drastically changed sockeye salmon ecology over the past two centuries and how this has affected IHNV evolution is unknown. The different ecology between salmonid species and environments offers interesting contrasts between viral evolution in rainbow trout aquaculture compared to declining wild and hatchery-managed sockeye populations, since they differ in factors such as temperature sensitivity and habitat condition [39,68]. These topics could not be fully explored here because the number of U isolates tested in this study was limited due to the focus on M genogroup evolution.

It is also possible that other mechanisms besides host ecology and aquaculture practices might have shaped IHNV M genogroup virulence evolution after emergence. Host specialism by the virus may be one such mechanism. Our data for the earliest viruses tested here, isolated in 1974 and 1976, show that following the host jump in the late 1960s, M genogroup IHNV very rapidly gained virulence in rainbow trout and lost virulence in sockeye salmon. Had a more gradual increase in virulence occurred, we would have expected more similar virulence measures among the oldest U and M isolates in both hosts. For IHNV, the specialization of U genogroup to sockeye and M genogroup to rainbow trout is well established in terms of field prevalence, fitness, and virulence [30,33,35,41,42,46,49,50,69]. Theory predicts that specialist pathogens will be more virulent than generalists [70,71]. We observed evidence of this here, in that ancestral U genogroup isolates were able to produce low levels of mortality in the novel rainbow trout host in addition to high virulence in sockeye, indicating they had a small amount of generalist capability. However, U genogroup isolates in sockeye were qualitatively less pathogenic than M isolates in rainbow trout. In contrast, the M genogroup appears to have a higher degree of host specialization. This is supported by our finding that in all but one case, M genogroup isolates were more virulent than U isolates in rainbow trout, and almost no mortality from M isolates was observed in the ancestral host. Similar patterns of viruses optimizing fitness at higher levels of virulence have been observed in a variety of systems through serial passage experiments [72].

In addition to studying how virulence evolved following a host jump, our experiments were also designed to test whether evolution of the IHNV M genogroup involved an adaptation to higher temperature. In wild environments, sockeye typically reside at seasonally fluctuating temperatures averaging ~10°C, whereas farmed rainbow trout in southern Idaho are maintained year-round at 15°C by a spring-fed aquifer [30,37]. Early after IHNV emergence in rainbow trout, epidemics were reported for some years at farms in other regions at lower temperatures (9–10°C), but no such reports originated at facilities that used higher temperatures (16–20°C) even though they were likely receiving contaminated eggs from the same source [36,73–76]. The eventual dramatic emergence and spread of IHNV in southern Idaho trout farms at 15°C between 1978–1980 [37] was therefore hypothesized to indicate an adaptation of M IHNV to the higher temperature, but there is little data to assess this idea as few empirical studies compare more than one temperature. In one study, one M genogroup strain had a higher virulence in rainbow trout at 15°C compared to 10°C, but this was not consistent with two other M strains in rainbow trout [41] or one M strain in steelhead [77]. Therefore the study presented here is novel in directly comparing virulence of multiple M isolates at both 10 and 15°C. Our results provide some support for the hypothesized temperature adaptation in that the predicted rate of virulence evolution across the date of viral isolate collection was greater at 15°C compared to 10°C (Fig 2). In other words, M isolates were on average more virulent at 15°C compared to 10°C (Fig 3), but the difference was smallest for the oldest isolates. As such, the virus appears to have undergone continued evolution through time, providing it with higher virulence at 15°C compared to 10°C, which was not as pronounced at the time of emergence. One pertinent question is whether the host or temperature adaptation happened first. If IHNV adapted to the novel host before increased temperature, in our study we would expect to see the older M isolates were more virulent at 10°C compared to 15°C, in rainbow trout. There was a suggestive pattern that this was true for the oldest isolate, HaVT74, but only at the highest exposure dosage and the effect was not statistically significant (Fig 2). However, the second oldest isolate (1976) showed higher virulence at 15°C compared to 10°C. We also note that on average U isolates were also more virulent at 15°C compared to 10°C in rainbow trout, although the effect was much smaller (Fig 4B). This may indicate a general increased rainbow trout susceptibility to IHNV at 15°C, regardless of genogroup. Collectively, our results suggests that if a temperature adaption occurred, it may have been very close to the time of the host jump, before most of the isolates in this study were collected [78], and it may not have been a major driver of adaptation of the virus to the new host. Characterization of the virulence-temperature interaction for additional M isolates collected immediately after the time of the host jump and their nearest U genogroup ancestors would be needed to resolve the timing and importance of the temperature adaptation, but it is unlikely that they are available. Regardless, M genogroup IHNV was significantly more virulent than U genogroup in rainbow trout regardless of temperature, indicating that a host adaptation occurred during emergence and not only a temperature adaptation.

The evolving IHNV-salmonid system continues to provide a valuable set of investigative resources for virus evolution research. A remaining mechanistic question is whether the evolution of increased virulence in emergent M genogroup IHNV was adaptive. The temporal movement towards dominance of high virulence M genogroup isolates in the field suggests that virulence is at least linked with other adaptive traits. A positive link between IHNV virulence and fitness traits such as viral replication and shedding is established in rainbow trout [4,43]. In particular, more virulent isolates appear to have longer shedding durations [4], suggesting a transmission duration advantage akin to that of myxoma virus virulence [8]. However, direct assessment of transmission for emergent M genogroup IHNV has not yet been conducted and whether increased virulence evolved via increased transmission rates as observed in other systems [13,15,57,79], remains to be determined. Likewise, investigating host-pathogen genomics and transcriptomics offers ripe opportunities for unravelling the genetic drivers of virulence.

These findings highlight the importance of understanding evolutionary trajectories and the diversity among viral phenotypes for effective pathogen management. Increasing virulence represents a major disease mitigation challenge, particularly given the rise in pathogen emergence events across systems [25,80,81]. Given that aquaculture is globally the most rapidly expanding sector of food production [82], understanding how its practices may drive virulence evolution of emergent pathogens is paramount for long-term disease management. For salmonids specifically, it presents a serious threat to fish production and natural biodiversity via risk of spillback events. Next steps include strategic consideration of how virulence evolution could be managed. Whether specific aquaculture practices or other aspects of rainbow trout and viral biology are the primary drivers shaping IHNV virulence evolution warrants further investigation. Control over stocking density, vaccination, selective breeding, and culling are tools that could be modified for curbing IHNV virulence in aquaculture [1,4], and also have relevance to a variety of agricultural and wildlife systems. Identification and integration of effective management may guide pathogen evolution away from the most damaging outcomes, thus safeguarding resources including essential food production, managed species, agriculture, and services provided by resilient natural ecosystems [3,15,80,83–86]. Gaining a comprehensive understanding of viral traits, host and environmental factors, and the strength of their relationships is critical for modeling the host-pathogen coevolutionary pathway, risk landscapes, and feasible management options.

## Methods

### Ethics statement

All research animals were handled in accordance with William & Mary Institutional Animal Care and Use Committee protocols (IACUC-2018-06-21-12998-arwargo and IACUC-2021-07-02-15072-arwargo).

1 Virus selection

Sixteen genetically unique IHNV isolates from the USGS archive [55] previously collected in the field, were used in this study (Table 1). The isolates were collected as part of on-going IHNV monitoring efforts by State, Federal, and Tribal agencies since the 1970s in response to disease events or as part of background surveillance efforts. The isolates were sent to USGS for genotyping, diagnostics, and archiving. All isolates were confirmed to be unique sequences via mid-glycoprotein gene (mid-G) sequencing as previously described [33,87]. Five U genogroup isolates were selected to represent dominant genotypes in the field of the ancestral lineage of IHNV which did not jump hosts [33,87]. Eleven M genogroup isolates were selected to span the temporal, spatial, and phylogenetic history of IHNV emergence and subsequent evolution in North American rainbow trout aquaculture. Both U and M genogroup isolates included viruses from three temporal bins, with collection dates between 1974–1977, 1990–1994, and 2012–2017. The isolates were propagated in EPC or CHSE-214 fish cell lines in Minimum Essential Media supplemented with 10% fetal bovine serum, 2mM L-glutamine, 50 units/mL penicillin, 50 µg/mL streptomycin, 20 µg/mL gentamycin, 2.5 µg/mL amphotericin B, and 0.15 mg/mL sodium bicarbonate (MEM-10) to generate viral stocks, which were titered by plaque assays independently at both the Virginia Institute of Marine Science (VIMS) and USGS Western Fisheries Research Center (WFRC) then stored at -80°C for later use [88,89].

2 Host species

To represent the ancestral IHNV host, sockeye salmon (*Oncorhynchus nerka*) were provided as eggs by Baker Lake Fish Hatchery (Washington Department of Fish and Wildlife, Washington, USA), produced as part of the state hatchery salmon conservation program. To represent the emergent IHNV host, rainbow trout (*O. mykiss*) eggs were obtained from a commercial trout egg producer. Neither the sockeye salmon nor rainbow trout lines are known to have undergone artificial selective breeding for IHNV resistance and are considered completely susceptible to infection. Trout eggs were produced from a minimum of twelve parental steelhead families (anadromous stocks of *O. mykiss*), and sockeye eggs were from a minimum of twelve females each fertilized with two males. All fish eggs were shipped directly to the respective research institutions (VIMS and WFRC), with the same cohort of fish used across locations. Eggs were iodine treated (10-minute soak in 1% solution) to inactivate IHNV and external pathogens and then reared in flow-through (2–4 tank exchanges/hour), specific pathogen-free, UV-irradiated fresh water maintained at 10°C or 12.5°C for sockeye and rainbow trout respectively. After hatching and complete digestion of yolk-sacs, fish fry were fed a standard trout diet (Zeigler- VIMS, Skretting - WFRC) at 2–4% body weight until they reached 1–2 grams of size when they were used for experiments. To acclimate sub-stocks to two different temperatures, rainbow trout were split into two identical tanks and water temperature gradually stepped up or down to 15 or 10°C over three weeks. Fish were then allowed to acclimate for a minimum of two weeks prior to the beginning of experiments. All sockeye experiments were conducted at 10°C, so acclimatization was not required.

3 *In vivo* challenge

We exposed triplicate batches of 20 fish (sockeye salmon or rainbow trout) to ancestral and derived viral isolates of IHNV (U and M genogroups respectively) at controlled doses (2x10^3^ or 2x10^5^ pfu/mL) and at a fixed temperature of 10°C in replicated experiments conducted at two locations [USGS Western Fisheries Research Center, Seattle, WA (WFRC) and Virginia Institute of Marine Science, William & Mary, Gloucester Point, VA (VIMS)]. For rainbow trout we also replicated this experiment at 15°C, which is the temperature used in the majority of rainbow trout aquaculture in the United States, and this treatment allows us to test for a theorized temperature adaptation. For all treatments we monitored fish daily for mortality and analyzed the results using generalized linear regression.

To initiate virulence assays, a standard *in vivo* batch immersion challenge method was used [41–44]. Briefly, triplicate groups of 15–20 fish were exposed to a High (2 x 10^5^ pfu/mL) or Low (2 x 10^3^ pfu/mL) dosage of each viral isolate (Table 1) diluted in MEM-10, or mock exposed to culture media, by adding 5 mL of inoculum to 995 mL of static water in 5 or 6 L tanks under aeration. Fish were held static for 1 hour, then maintained on aerated flow-through water (~150 mL/min), until mortality plateaued (28–56 days, Fig 1). Sockeye were monitored for longer than rainbow trout due to known slower mortality kinetics [41,42]. Mortality was recorded and dead fish were removed from tanks, daily. The experiments were separated into three blocks: sockeye at 10°C, rainbow trout at 10°C, and rainbow trout at 15°C. These were replicated at both the VIMS and WFRC labs, for a total of 6 independent experiments. Rainbow trout experiments at the two temperatures were conducted within 1–2 weeks of each other to control for age (degree-days) and size (1.52 ± 0.36 grams).

4 Statistical analysis

Statistical tests and visualizations were carried out in R Statistical Software (version 4.2.3) [90] and RStudio (version 2023.12.1 + 402) [91]. All data was analyzed using generalized linear models with a binomial error structure (“lme4” and “stats” packages) to elucidate virulence differences between treatments, measured as the total number of dead and live fish at the end of the experiment (i.e., logistic regression on cumulative probability of fish death) [90,92]. The analysis was then broken into three parts.

1. To investigate how IHNV virulence evolved in the emergent M genogroup since the host jump, data from only the M isolates in rainbow trout were analyzed. Dose (2000 vs 200000 pfu/mL – categorical), temperature (10 vs 15°C – categorical) and year of virus isolate collection (continuous) centered around the year 2000, were included as fixed effects. Location of experiment (VIMS or WFRC), isolate (see Table 1), and tank [replicates 1–3] were included (all categorical) as random effects. Due to model convergence issues caused by some groups having 0% mortality, one imaginary “dead” fish and one imaginary “alive” fish were added to the data from each tank [93]. This adjustment made the analysis more conservative (less likely to see statistical differences) because each treatment was pushed slightly towards 50% mortality. In addition to indicating how M genogroup virulence has changed as a function of isolate collection date, this analysis also made it possible to directly compare the virulence of each isolate. For this part of the analysis, isolate (categorical) was included as a fixed effect and location of the experiment was included as a random effect, with only data from the most environmentally critical temperature (15°C). Year of collection and tank were explored as additional random effects but resulted in overfitting, and therefore were not included in the final analysis.
2. A similar approach was used to compare differences between M and U genogroup virus, including all data. The two host species were analyzed separately, to account for independent experiments and different temperature treatments. For both hosts, factors in the model were the same as analysis 1 with the addition of genogroup as a fixed factor (U or M – categorical) and the removal of the year factor. Temperature was dropped for the sockeye since only one temperature treatment (10°C) is environmentally relevant and was conducted. Including all random effects resulted in overfitting for sockeye, so location and tank were dropped from that analysis.
3. To compare the virulence of ancestral isolates in the ancestral host, the analysis focused exclusively on data from U isolates in sockeye salmon, with isolate name (categorical) and dose included as fixed factors. Models with the random effects experiment and tank did not converge or were deemed a poor fit relative to other models, so the terms were dropped. The GLM model from the “stats” package (version 4.2.3) function was then employed to allow for exclusion of all random terms [90].

For all analyses, model selection was conducted using corrected Akaike Information Criterion (AICc), where maximal models (i.e., those containing all main effects and interactions) were fit to the data and the dredge function from the “MuMIn” package (version 1.47.5) was used to identify the lowest AICc value from all possible combinations [94]. Results of the best model according to AICc (model with lowest AICc) are presented in the main text and alternate models ranked by AICc are shown in the supplementary materials. Because AICc selection was used, p-values are not provided, and instead ΔAICc values for the model without the factor of discussion is presented in the results. Coefficients with standard error from summaries of best fit models, as well as plotting of predicted values, were used to show the magnitude and direction of factor level differences for best fit models. The predicted probability of fish death and 95% confidence intervals were calculated using the predictSE function from the “AICcmodavg” package (version 2.3-3) and multiplying the standard error by 1.96 (assuming a normal distribution of the population variance), for the factors of interest in the AICc selected models [95]. In the main body text, this is presented in an additive way, showing the percent increase in the odds of death due to the factor level of interest [(*e*^coefficient value^ - 1) x 100%)], compared to the baseline. In cases of interaction terms or where factors contained more than two levels post-hoc pairwise comparisons of the estimated marginal means were performed with the “emmeans” package (version 1.8.8) [96], using a Tukey correction for multiple tests to determine significant differences between factor levels.

## Supporting information

S1 Fig Cumulative survival data from virulence assays not shown in Fig 1. Panels show mean cumulative proportion survival through time, for triplicate tanks in each experimental treatment. Tanks contained 20 fish each, except for experiment 3 which contained 15 fish each. (A) Experiment 2; sockeye held at 10°C at WFRC. (B) Experiment 6; rainbow trout held at 15°C at WFRC. (C) Experiment 4; rainbow trout held at 10°C at WFRC. (D) Experiment 3; rainbow trout held at 10°C at VIMS. For all panels, solid lines indicate high dose (2 x 10^5^ pfu/mL); dashed lines indicate low dose (2 x 10^3^ pfu/mL) virus exposure. Standard error (± 1) between the triplicate tanks is indicated by a shaded ribbon. Treatments that were not included in an experiment are marked ND. Treatment plots are ordered by IHNV genogroup (U top row, M bottom rows), followed by year of isolation and isolate name. Mortality was tracked for longer in sockeye experiments so x-axis scales are different (panel A).
(TIF)

S1 Table GLME model output for analysis of M isolate virulence evolution over time since host jumping to rainbow trout. Estimates and associated error are on logit scale. The degrees of freedom for residuals were 365. Odds-ratio estimates were obtained with the formula e^(logit value)^.
(DOCX)

S2 Table GLME candidate models for analysis of M isolate virulence evolution over time since host jumping to rainbow trout. Model 1 is the best-fit model reported. A ‘+’ indicates whether or not the main effect was included in the respective model. See S1 Table for coefficients top model.
(DOCX)

S3 Table GLME model output for analysis of M isolate variation in virulence following low dose exposure (2 x 10^3^ pfu/mL) at 15°C. Coefficient estimates for each isolate and associated error are on logit scale. Corresponding odds-ratio estimates were obtained with the formula e^(logit value)^, compared to baseline isolate HaVT74. Total degrees of freedom for residuals in the model were 51.
(DOCX)

S4 Table Pairwise comparisons for analysis of M isolate variation in virulence following low dose exposure (2 x 10^3^ pfu/mL) at 15°C. Estimated marginal means are reported using a Tukey correction for multiple tests. See S3 Table for coefficients top model.
(DOCX)

S5 Table GLME model output for analysis of M isolate variation in virulence following high dose exposure (2 x 10^5^ pfu/mL) at 15°C. Coefficient estimates for each isolate and associated error are on logit scale. Corresponding odds-ratio estimates were obtained with the formula e^(logit value)^, with isolate HaVT74 set as baseline. Total degrees of freedom for residuals in the model were 51.
(DOCX)

S6 Table Pairwise comparisons for analysis of M isolate variation in virulence following high dose exposure (2 x 10^5^ pfu/mL) at 15°C. Estimated marginal means are reported using a Tukey correction for multiple tests. See S5 Table for coefficients top model.
(DOCX)

S7 Table Model summary comparing U versus M virulence in sockeye hosts. Estimates and associated error are on logit scale. Corresponding odds-ratio estimates were obtained with the formula e^(logit value)^. Residual degrees of freedom = 170.
(DOCX)

S8 Table Candidate models for comparing U versus M virulence in sockeye hosts. Model 1 is the best-fit model. A ‘+’ indicates whether or not the main effect was included in the respective model. See S7 Table for top model coefficients.
(DOCX)

S9 Table Model summary for comparing U versus M virulence in rainbow trout hosts. Estimates and associated error are on logit scale. Corresponding odds-ratio estimates were obtained with the formula e^(logit value)^. The degrees of freedom for residuals were 170.
(DOCX)

S10 Table GLME candidate models for comparing U versus M virulence in rainbow trout hosts. Model 1 is the best-fit model. A ‘+’ indicates whether the main effect was included in the respective model. See S9 Table for top model coefficients.
(DOCX)

S11 Table GLM model output for U isolate variation in virulence. Coefficient estimates for each isolate and associated error are on logit scale. Corresponding odds-ratio estimates were obtained with the formula e^(logit value)^. Residual degrees of freedom = 54.
(DOCX)

S12 Table GLM candidate models for examining U isolate variation in virulence. Model 1 is the best-fit model. A ‘+’ indicates whether the main effect was included in the respective model. See S11 Table for top model coefficients.
(DOCX)
