# Supplementary material for: Virulence evolution of a salmonid virus following a host jump
Source: PLoS Pathog. 2025 Dec 17;21(12):e1013806. doi: 10.1371/journal.ppat.1013806 (PMC12721516; doi:10.1371/journal.ppat.1013806)
Supplement: S1 Table — Estimates and associated error are on logit scale. The degrees of freedom for residuals were 365. Odds-ratio estimates were obtained with the formula e(logit value). (DOCX) [file ppat.1013806.s002.docx]

**Table S1. GLME model output for analysis of M isolate virulence evolution over time since host jumping to rainbow trout.** Estimates and associated error are on logit scale. The degrees of freedom for residuals were 365. Odds-ratio estimates were obtained with the formula e^(logit value)^.

| **Fixed effect** | **Estimate (logit)** | **Standard error (logit)** | **Estimate (odds ratio)** | **Z-value** | **Degrees of freedom** |
| --- | --- | --- | --- | --- | --- |
| Intercept | -0.655611 | 0.364417 | 0.519 | -1.799 |  |
| Year of Isolation | 0.023017 | 0.013510 | 1.023 | 1.704 | 6 |
| Dose (High) | 1.599523 | 0.146394 | 4.951 | 10.926 | 1 |
| Temperature (15℃) | 0.918181 | 0.142626 | 2.505 | 6.438 | 1 |
| Year*Temp | 0.011591 | 0.006586 | 1.012 | 1.760 | 13 |
| Dose*Temp | -0.414032 | 0.207136 | 0.661 | -1.999 | 3 |
| Model: cbind(Dead, Alive) ~ (1\|Lab) + (1\|Tank) + (1\|Isolate) + CenterYr + Dose + Temp + CenterYr:Temp + Dose:Temp, family="binomial" | | | | | |
