# Supplementary material for: Virulence evolution of a salmonid virus following a host jump
Source: PLoS Pathog. 2025 Dec 17;21(12):e1013806. doi: 10.1371/journal.ppat.1013806 (PMC12721516; doi:10.1371/journal.ppat.1013806)
Supplement: S2 Table — Model 1 is the best-fit model reported. A ‘+’ indicates whether or not the main effect was included in the respective model. See S1 Table for coefficients top model. (DOCX) [file ppat.1013806.s003.docx]

**Table S2. GLME candidate models for analysis of M isolate virulence evolution over time since host jumping to rainbow trout.** Model 1 is the best-fit model reported. A ‘+’ indicates whether or not the main effect was included in the respective model. See Table S1 for coefficients top model.

| Model | Collection Year | Dose | Temp | Year* Dose | Year* Temp | Dose* Temp | Year* Dose* Temp | df | ΔAICc | AICc weight |
| --- | --- | --- | --- | --- | --- | --- | --- | --- | --- | --- |
| 1 | 0.023 | + | + |  | + | + |  | 9 | 0.00 | 0.276 |
| 2 | 0.029 | + | + |  |  | + |  | 8 | 0.90 | 0.176 |
| 3 | 0.023 | + | + |  | + |  |  | 8 | 1.78 | 0.114 |
| 4 | 0.025 | + | + | + | + | + |  | 10 | 1.89 | 0.107 |
| 5 |  | + | + |  |  | + |  | 7 | 2.70 | 0.071 |
| 6 | 0.031 | + | + | + |  | + |  | 9 | 2.72 | 0.071 |
| 7 | 0.029 | + | + |  |  |  |  | 7 | 3.09 | 0.059 |
| 8 | 0.024 | + | + | + | + |  |  | 9 | 3.71 | 0.043 |
| 9 | 0.025 | + | + | + | + | + | + | 11 | 4.08 | 0.036 |
| 10 |  | + | + |  |  |  |  | 6 | 4.93 | 0.023 |
