# Supplementary material for: Virulence evolution of a salmonid virus following a host jump
Source: PLoS Pathog. 2025 Dec 17;21(12):e1013806. doi: 10.1371/journal.ppat.1013806 (PMC12721516; doi:10.1371/journal.ppat.1013806)
Supplement: S3 Table — Coefficient estimates for each isolate and associated error are on logit scale. Corresponding odds-ratio estimates were obtained with the formula e(logit value), compared to baseline isolate HaVT74. Total degrees of freedom for residuals in the model were 51. (DOCX) [file ppat.1013806.s004.docx]

**Table S3. GLME model output for analysis of M isolate variation in virulence following low dose exposure (2 x 10^3^ pfu/mL) at 15°C.** Coefficient estimates for each isolate and associated error are on logit scale. Corresponding odds-ratio estimates were obtained with the formula e^(logit value)^, compared to baseline isolate HaVT74. Total degrees of freedom for residuals in the model were 51.

| **Coefficient** | **Estimate (logit)** | **Standard error (logit)** | **Estimate (odds-ratio)** | **Z-value** |
| --- | --- | --- | --- | --- |
| Intercept | -0.7825 | 0.7637 | 0.457 | -1.025 |
| Isolate (SV76) | -0.6931 | 0.3165 | 0.500 | -2.190 |
| Isolate (220-90) | 1.7098 | 0.3090 | 5.278 | 5.534 |
| Isolate (Ha20-91) | 0.5238 | 0.2988 | 1.688 | 1.753 |
| Isolate (Ha30-91) | 1.1717 | 0.3015 | 3.228 | 3.887 |
| Isolate (Ha39-91) | 0.4459 | 0.2995 | 1.562 | 1.489 |
| Isolate (Ht508K-14) | 2.3688 | 0.3271 | 10.685 | 7.242 |
| Isolate (Ht511-14) | 1.2154 | 0.3019 | 3.372 | 4.026 |
| Isolate (HtBrG-16) | 0.4714 | 0.3557 | 1.602 | 1.325 |
| Isolate (HtBrK-16) | 2.3688 | 0.3271 | 10.685 | 7.242 |
| Isolate (Ht134-17) | 1.8518 | 0.3119 | 6.371 | 5.937 |
| Model: cbind(Dead,Alive) ~ (1\|Lab) + Isolate, family="binomial" | | | | |
