# Supplementary material for: Virulence evolution of a salmonid virus following a host jump
Source: PLoS Pathog. 2025 Dec 17;21(12):e1013806. doi: 10.1371/journal.ppat.1013806 (PMC12721516; doi:10.1371/journal.ppat.1013806)
Supplement: S5 Table — Coefficient estimates for each isolate and associated error are on logit scale. Corresponding odds-ratio estimates were obtained with the formula e(logit value), with isolate HaVT74 set as baseline. Total degrees of freedom for residuals in the model were 51. (DOCX) [file ppat.1013806.s006.docx]

**Table S5. GLME model output for analysis of M isolate variation in virulence following high dose exposure (2 x 10^5^ pfu/mL) at 15°C.** Coefficient estimates for each isolate and associated error are on logit scale. Corresponding odds-ratio estimates were obtained with the formula e^(logit value)^, with isolate HaVT74 set as baseline. Total degrees of freedom for residuals in the model were 51.

| **Coefficient** | **Estimate (logit)** | **Standard error (logit)** | **Estimate (odds-ratio)** | **Z-value** |
| --- | --- | --- | --- | --- |
| Intercept | 0.9653 | 0.7498 | 2.626 | 1.287 |
| Isolate (SV76) | -1.4995 | 0.3087 | 0.223 | -4.857 |
| Isolate (220-90) | 1.1847 | 0.3492 | 3.270 | 3.393 |
| Isolate (Ha20-91) | 0.1909 | 0.3089 | 1.210 | 0.618 |
| Isolate (Ha30-91) | 1.7130 | 0.3914 | 5.546 | 4.377 |
| Isolate (Ha39-91) | 0.5523 | 0.3190 | 1.737 | 1.732 |
| Isolate (Ht508K-14) | 1.5157 | 0.3735 | 4.552 | 4.058 |
| Isolate (Ht511-14) | 0.2852 | 0.3115 | 1.330 | 0.916 |
| Isolate (HtBrG-16) | 0.9826 | 0.6436 | 2.671 | 1.527 |
| Isolate (HtBrK-16) | 2.5885 | 0.5113 | 13.310 | 5.063 |
| Isolate (Ht134-17) | 2.3921 | 0.4777 | 10.936 | 5.007 |
| Model: cbind(Dead,Alive) ~ (1\|Lab) + Isolate, family="binomial" | | | | |
