# Supplementary material for: Virulence evolution of a salmonid virus following a host jump
Source: PLoS Pathog. 2025 Dec 17;21(12):e1013806. doi: 10.1371/journal.ppat.1013806 (PMC12721516; doi:10.1371/journal.ppat.1013806)
Supplement: S6 Table — Estimated marginal means are reported using a Tukey correction for multiple tests. See S5 Table for coefficients top model. (DOCX) [file ppat.1013806.s007.docx]

**Table S6. Pairwise comparisons for analysis of M isolate variation in virulence following high dose exposure (2 x 10^5^ pfu/mL) at 15°C .** Estimated marginal means are reported using a Tukey correction for multiple tests. See Table S5 for coefficients top model.

| Contrast | Estimate | SE | Z-ratio | p-value |
| --- | --- | --- | --- | --- |
| HaVT74 - (220-90) | -1.185 | 0.349 | -3.393 | 0.029 |
| HaVT74 - (Ha20-91) | -0.191 | 0.309 | -0.618 | 1.000 |
| HaVT74 - (Ha30-91) | -1.713 | 0.391 | -4.377 | 0.001 |
| HaVT74 - (Ha39-91) | -0.552 | 0.319 | -1.732 | 0.819 |
| HaVT74 - (Ht134-17) | -2.392 | 0.478 | -5.007 | 0.000 |
| HaVT74 - (Ht508K-14) | -1.516 | 0.373 | -4.058 | 0.002 |
| HaVT74 - (Ht511-14) | -0.285 | 0.312 | -0.916 | 0.998 |
| HaVT74 - (HtBrG-16) | -0.983 | 0.644 | -1.527 | 0.911 |
| HaVT74 - (HtBrK-16) | -2.589 | 0.511 | -5.063 | 0.000 |
| HaVT74 - SV76 | 1.500 | 0.309 | 4.857 | 0.000 |
| (220-90) - (Ha20-91) | 0.994 | 0.352 | 2.824 | 0.149 |
| (220-90) - (Ha30-91) | -0.528 | 0.425 | -1.244 | 0.977 |
| (220-90) - (Ha39-91) | 0.632 | 0.360 | 1.756 | 0.806 |
| (220-90) - (Ht134-17) | -1.207 | 0.505 | -2.389 | 0.373 |
| (220-90) - (Ht508K-14) | -0.331 | 0.408 | -0.810 | 0.999 |
| (220-90) - (Ht511-14) | 0.900 | 0.354 | 2.541 | 0.281 |
| (220-90) - (HtBrG-16) | 0.202 | 0.671 | 0.301 | 1.000 |
| (220-90) - (HtBrK-16) | -1.404 | 0.537 | -2.614 | 0.241 |
| (220-90) - SV76 | 2.684 | 0.357 | 7.527 | 0.000 |
| (Ha20-91) - (Ha30-91) | -1.522 | 0.394 | -3.865 | 0.005 |
| (Ha20-91) - (Ha39-91) | -0.361 | 0.322 | -1.122 | 0.990 |
| (Ha20-91) - (Ht134-17) | -2.201 | 0.480 | -4.589 | 0.000 |
| (Ha20-91) - (Ht508K-14) | -1.325 | 0.376 | -3.523 | 0.019 |
| (Ha20-91) - (Ht511-14) | -0.094 | 0.315 | -0.300 | 1.000 |
| (Ha20-91) - (HtBrG-16) | -0.792 | 0.647 | -1.225 | 0.980 |
| (Ha20-91) - (HtBrK-16) | -2.398 | 0.513 | -4.673 | 0.000 |
| (Ha20-91) - SV76 | 1.690 | 0.313 | 5.392 | 0.000 |
| (Ha30-91) - (Ha39-91) | 1.161 | 0.401 | 2.894 | 0.125 |
| (Ha30-91) - (Ht134-17) | -0.679 | 0.535 | -1.269 | 0.974 |
| (Ha30-91) - (Ht508K-14) | 0.197 | 0.445 | 0.444 | 1.000 |
| (Ha30-91) - (Ht511-14) | 1.428 | 0.396 | 3.609 | 0.014 |
| (Ha30-91) - (HtBrG-16) | 0.730 | 0.694 | 1.052 | 0.994 |
| (Ha30-91) - (HtBrK-16) | -0.875 | 0.565 | -1.549 | 0.903 |
| (Ha30-91) - SV76 | 3.213 | 0.399 | 8.056 | 0.000 |
| (Ha39-91) - (Ht134-17) | -1.840 | 0.486 | -3.789 | 0.007 |
| (Ha39-91) - (Ht508K-14) | -0.963 | 0.384 | -2.511 | 0.298 |
| (Ha39-91) - (Ht511-14) | 0.267 | 0.324 | 0.823 | 0.999 |
| (Ha39-91) - (HtBrG-16) | -0.430 | 0.653 | -0.659 | 1.000 |
| (Ha39-91) - (HtBrK-16) | -2.036 | 0.519 | -3.926 | 0.004 |
| (Ha39-91) - SV76 | 2.052 | 0.325 | 6.312 | 0.000 |
| (Ht134-17) - (Ht508K-14) | 0.876 | 0.522 | 1.678 | 0.847 |
| (Ht134-17) - (Ht511-14) | 2.107 | 0.481 | 4.379 | 0.001 |
| (Ht134-17) - (HtBrG-16) | 1.409 | 0.747 | 1.887 | 0.726 |
| (Ht134-17) - (HtBrK-16) | -0.196 | 0.628 | -0.313 | 1.000 |
| (Ht134-17) - SV76 | 3.892 | 0.484 | 8.036 | 0.000 |
| (Ht508K-14) - (Ht511-14) | 1.230 | 0.378 | 3.256 | 0.045 |
| (Ht508K-14) - (HtBrG-16) | 0.533 | 0.684 | 0.779 | 1.000 |
| (Ht508K-14) - (HtBrK-16) | -1.073 | 0.553 | -1.940 | 0.691 |
| (Ht508K-14) - SV76 | 3.015 | 0.381 | 7.915 | 0.000 |
| (Ht511-14) - (HtBrG-16) | -0.697 | 0.648 | -1.075 | 0.993 |
| (Ht511-14) - (HtBrK-16) | -2.303 | 0.514 | -4.478 | 0.000 |
| (Ht511-14) - SV76 | 1.785 | 0.317 | 5.636 | 0.000 |
| (HtBrG-16) - (HtBrK-16) | -1.606 | 0.769 | -2.088 | 0.586 |
| (HtBrG-16) - SV76 | 2.482 | 0.632 | 3.930 | 0.004 |
| (HtBrK-16) - SV76 | 4.088 | 0.517 | 7.901 | 0.000 |
