# Supplementary material for: Virulence evolution of a salmonid virus following a host jump
Source: PLoS Pathog. 2025 Dec 17;21(12):e1013806. doi: 10.1371/journal.ppat.1013806 (PMC12721516; doi:10.1371/journal.ppat.1013806)
Supplement: S7 Table — Estimates and associated error are on logit scale. Corresponding odds-ratio estimates were obtained with the formula e(logit value). Residual degrees of freedom = 170. (DOCX) [file ppat.1013806.s008.docx]

**Table S7. Model summary comparing U versus M virulence in sockeye hosts.** Estimates and associated error are on logit scale. Corresponding odds-ratio estimates were obtained with the formula e^(logit value)^. Residual degrees of freedom = 170.

| **Fixed effect** | **Estimate (logit)** | **Standard error (logit)** | **Estimate (odds-ratio)** | **Z-value** | **Degrees of freedom** |
| --- | --- | --- | --- | --- | --- |
| Intercept | -4.6355 | 0.3219 | 0.010 | -14.401 |  |
| Genogroup (U) | 3.7627 | 0.4501 | 43.065 | 8.359 | 1 |
| Dose (High) | 1.0772 | 0.1371 | 2.937 | 7.859 | 1 |
| Model: cbind(Dead, Alive) ~ (1\|Isolate) + Genogroup + Dose, family="binomial" | | | | | |
