# Supplementary material for: Virulence evolution of a salmonid virus following a host jump
Source: PLoS Pathog. 2025 Dec 17;21(12):e1013806. doi: 10.1371/journal.ppat.1013806 (PMC12721516; doi:10.1371/journal.ppat.1013806)
Supplement: S8 Table — Model 1 is the best-fit model. A ‘+’ indicates whether or not the main effect was included in the respective model. See S7 Table for top model coefficients. (DOCX) [file ppat.1013806.s009.docx]

**Table S8. Candidate models for comparing U versus M virulence in sockeye hosts.** Model 1 is the best-fit model. A ‘+’ indicates whether or not the main effect was included in the respective model. See Table S7 for top model coefficients.

| Model | Dose | Genogroup | Dose* Genogroup | df | ΔAICc | AICc weight |
| --- | --- | --- | --- | --- | --- | --- |
| 1 | + | + |  | 4 | 0.00 | 0.694 |
| 2 | + | + | + | 5 | 1.63 | 0.306 |
| 3 | + |  |  | 3 | 22.58 | 0.000 |
| 4 |  | + |  | 3 | 62.78 | 0.000 |
| 5 |  |  |  | 2 | 85.25 | 0.000 |
