# Supplementary material for: Virulence evolution of a salmonid virus following a host jump
Source: PLoS Pathog. 2025 Dec 17;21(12):e1013806. doi: 10.1371/journal.ppat.1013806 (PMC12721516; doi:10.1371/journal.ppat.1013806)
Supplement: S9 Table — Estimates and associated error are on logit scale. Corresponding odds-ratio estimates were obtained with the formula e(logit value). The degrees of freedom for residuals were 170. (DOCX) [file ppat.1013806.s010.docx]

**Table S9. Model summary for comparing U versus M virulence in rainbow trout hosts.** Estimates and associated error are on logit scale. Corresponding odds-ratio estimates were obtained with the formula e^(logit value)^. The degrees of freedom for residuals were 170.

| **Fixed effect** | **Estimate (logit)** | **Standard error (logit)** | **Estimate (odds-ratio**) | **Z-value** | **Degrees of freedom** |
| --- | --- | --- | --- | --- | --- |
| Intercept | -3.3091 | 0.5637 | 0.037 | -5.871 |  |
| Genogroup (M) | 2.6673 | 0.4855 | 14.401 | 5.494 | 1 |
| Dose (High) | 1.6391 | 0.1165 | 5.151 | 14.070 | 1 |
| Temperature (15℃) | 0.8354 | 0.1146 | 2.306 | 7.287 | 1 |
| Model: cbind(Dead, Alive) ~ (1\|Isolate) + (1\|Tank) +(1\|Lab) + Genogroup + Dose + Temp, family="binomial" | | | | | |
