# Supplementary material for: Virulence evolution of a salmonid virus following a host jump
Source: PLoS Pathog. 2025 Dec 17;21(12):e1013806. doi: 10.1371/journal.ppat.1013806 (PMC12721516; doi:10.1371/journal.ppat.1013806)
Supplement: S10 Table — Model 1 is the best-fit model. A ‘+’ indicates whether the main effect was included in the respective model. See S9 Table for top model coefficients. (DOCX) [file ppat.1013806.s011.docx]

**Table S10. GLME candidate models for comparing U versus M virulence in rainbow trout hosts.** Model 1 is the best-fit model. A ‘+’ indicates whether the main effect was included in the respective model. See Table S9 for top model coefficients.

| Model | Dose | Genogroup | Temp | Genogroup* Temp | df | ΔAICc | AICc weight |
| --- | --- | --- | --- | --- | --- | --- | --- |
| 1 | + | + | + |  | 7 | 0.00 | 0.701 |
| 2 | + | + | + | + | 8 | 1.71 | 0.298 |
| 3 | + |  | + |  | 6 | 14.69 | 0.000 |
| 4 | + | + |  |  | 6 | 47.83 | 0.000 |
| 5 | + |  |  |  | 5 | 62.56 | 0.000 |
| 6 |  | + | + |  | 6 | 164.88 | 0.000 |
| 7 |  | + | + | + | 7 | 166.43 | 0.000 |
| 8 |  |  | + |  | 5 | 179.73 | 0.000 |
| 9 |  | + |  |  | 5 | 193.78 | 0.000 |
| 10 |  |  |  |  | 4 | 208.71 | 0.000 |
