# Supplementary material for: Virulence evolution of a salmonid virus following a host jump
Source: PLoS Pathog. 2025 Dec 17;21(12):e1013806. doi: 10.1371/journal.ppat.1013806 (PMC12721516; doi:10.1371/journal.ppat.1013806)
Supplement: S11 Table — Coefficient estimates for each isolate and associated error are on logit scale. Corresponding odds-ratio estimates were obtained with the formula e(logit value). Residual degrees of freedom = 54. (DOCX) [file ppat.1013806.s012.docx]

**Table S11. GLM model output for U isolate variation in virulence.** Coefficient estimates for each isolate and associated error are on logit scale. Corresponding odds-ratio estimates were obtained with the formula e^(logit value)^. Residual degrees of freedom = 54.

| **Coefficient** | **Estimate (logit)** | **Standard error** | **Estimate (odds-ratio)** | **Z-value** | **Degrees of freedom** |
| --- | --- | --- | --- | --- | --- |
| Intercept | -2.0183 | 0.2163 | 0.133 | -9.333 |  |
| Isolate (Blk15) | 1.2055 | 0.2502 | 3.338 | 4.817 | 4 |
| Isolate (Blk94) | 1.8706 | 0.2514 | 6.492 | 7.442 | 4 |
| Isolate (GF77) | 0.6967 | 0.2541 | 2.007 | 2.742 | 4 |
| Isolate (Wck74) | 1.8211 | 0.2509 | 6.179 | 7.259 | 4 |
| Dose (High) | 1.1210 | 0.1492 | 3.068 | 7.514 | 1 |
| Model: cbind(Dead,Alive) ~ Isolate + Dose, family="binomial" | | | | | |
