# Supplementary material for: Virulence evolution of a salmonid virus following a host jump
Source: PLoS Pathog. 2025 Dec 17;21(12):e1013806. doi: 10.1371/journal.ppat.1013806 (PMC12721516; doi:10.1371/journal.ppat.1013806)
Supplement: S12 Table — Model 1 is the best-fit model. A ‘+’ indicates whether the main effect was included in the respective model. See S11 Table for top model coefficients. (DOCX) [file ppat.1013806.s013.docx]

**Table S12. GLM candidate models for examining U isolate variation in virulence.** Model 1 is the best-fit model. A ‘+’ indicates whether the main effect was included in the respective model. See Table S11 for top model coefficients.

| Model | Dose | Isolate | Dose*Isolate | df | ΔAICc | AICc weight |
| --- | --- | --- | --- | --- | --- | --- |
| 1 | + | + |  | 6 | 0.00 | 0.985 |
| 2 | + | + | + | 10 | 8.34 | 0.015 |
| 3 |  | + |  | 5 | 56.82 | 0.000 |
| 4 | + |  |  | 2 | 80.25 | 0.000 |
| 5 |  |  |  | 1 | 133.48 | 0.000 |
